# Effect of Shenling Baizhu San on Intestinal Flora in a Rat Model of Ulcerative Colitis with Spleen Deficiency and Dampness

**DOI:** 10.1155/2022/9985147

**Published:** 2022-02-12

**Authors:** Yiting Luo, Fangyuan Zhu, Jiaqian Wu, Jin Wu, Pei Wu, Yingchao Liu

**Affiliations:** ^1^The Second Clinical Medical College, Zhejiang Chinese Medical University, Hangzhou 310053, China; ^2^Zhejiang Chinese Medical University, Hangzhou 310053, China

## Abstract

**Objective:**

Shenling Baizhu San (SLBZS) is reported as an effective drug for ulcerative colitis (UC); however, its effect on intestinal flora remains unknown. In this study, we investigated the effect of SLBZS on intestinal flora in a rat model of UC with spleen deficiency and dampness.

**Methods:**

UC was induced in rats using 2,4,6-trinitrobenzene sulfonic acid on the basis of a model of spleen deficiency and dampness. The 16S rDNA sequencing was used to detect structural changes in the intestinal flora; the phylogenetic investigation of communities by reconstruction of unobserved state (PICRUSt) analysis was used to predict the altered pathways.

**Results:**

Compared with the model group, rats in the SLBZS group exhibited decreased levels of TNF-*α*(*P* < 0.05), and increased abundance and diversity of the intestinal flora. The abundance of Actinobacteria (*P* < 0.001) and Bacteroides (*P* < 0.01) increased and that of Firmicutes decreased (*P* < 0.001), and the abundance of *Bifidobacterium*(*P* < 0.05) and *Allobaculum* increased. PICRUSt analysis showed that the altered pathways between the groups were those of fatty acid and antibiotic biosynthesis, amino acid metabolism, and the pentose phosphate pathway.

**Conclusions:**

SLBZS can regulate the structure and function of the intestinal flora, alter expression levels of certain metabolic pathways, and has the potential to treat UC.

## 1. Introduction

Ulcerative colitis (UC) is a chronic, nonspecific inflammatory disease that affects the colorectal mucosa. The main clinical manifestations of UC are diarrhea and bloody mucus in stools. The pathogenesis of UC remains unclear but is generally believed to involve environmental factors, genetic susceptibility, immune status, intestinal flora, and intestinal barrier function [1–3].

At present, the treatment of patients with UC is mainly based on Western medicine and includes the use of 5-aminosalicylic acid preparations, cortisol hormones, immunosuppressants, and biological agents, which induce and maintain clinical remission of UC. However, the rate of disease recurrence among patients is high, and there is a risk of serious infections and tumor development [4]. Treating UC with traditional Chinese medicine (TCM) has the unique advantage of providing comprehensive management, which can alleviate the condition and safely reduce recurrence, while causing fewer side effects [5, 6].

The theory of TCM divides UC into multiple types of syndromes, among which the syndrome of spleen deficiency and dampness is clinically one of the common syndromes [7] and believes that the spleen governs the transportation and transformation of water and dampness, while the deficiency of the spleen leads to a low transportation and transformation function, and clinical symptoms such as anorexia, loose stools, weight loss, and thick and greasy tongue coating will occur. Deficiency of the spleen is prone to dampness, dampness is easy to trap the spleen, and the spleen has a special sensitivity to dampness [8]. Multiple clinical syndrome research fund spleen deficiency is the root cause of UC, and dampness is the indicator of the disease. Whether it is initial or recurrent UC, spleen deficiency and dampness are more common, and they are mutually cause and effect. The theory of TCM believes that the occurrence of spleen deficiency and dampness syndrome is generally closely related to factors such as exogenous dampness, eating disorder, fatigue, internal injury, or emotional disorder [9]. Shenling Baizhu San (SLBZS), a prescription from the “Taiping Huimin Heji Bureau Prescription” in the Song dynasty, is a commonly used prescription for UC with spleen deficiency and dampness in clinical practice. Studies showed that compared with mesalazine in the treatment of UC, SLBZS is significantly better in terms of clinical efficacy, intestinal mucosal changes under colonoscopy, and occurrence of adverse reactions [10, 11]. Some studies showed that the total effective rate of patients who were prescribed SLBZS combined with mesalazine in the treatment of UC was 93.02% and that of patients who used mesalazine alone was 76.74%. Patients treated with SLBZS combined with mesalazine had lower serum inflammatory factor levels, and the occurrence of adverse reactions was lower [12]. These researchers all expressed the opinion that SLBZS is worthy of application and promotion. In recent years, research has shown that changes in intestinal flora play an important role in the occurrence and development of UC [13, 14]. Therefore, it is of great significance to study the relationship between SLBZS and intestinal flora in UC.

The aim of this study is to explore the effects of SLBZS on the structure and function of intestinal flora in a rat model of UC with spleen deficiency and dampness. In this experiment, a multifactor “combination of disease and syndrome” modeling method and 2,4,6-trinitrobenzene sulfonic (TNBS) acid induction along with environment and diet intervention will be used to establish a rat model of UC with spleen deficiency and dampness. We will use the 16S rDNA high-throughput sequencing technology and the phylogenetic investigation of communities by reconstruction of unobserved state (PICRUSt) analysis to explore the efficacy of SLBZS in the treatment of UC by examining changes in the structure and function of the intestinal flora and the mechanism of action in regard to the intestinal flora.

## 2. Materials and Methods

### 2.1. Animals

Forty-five clean-grade male Sprague Dawley rats, weighing 250–300 g, were purchased from Shanghai SLAC Laboratory Animal Co., Ltd., and the experimental animal production license number was SCXK (Shanghai) 2017–0005. The rats were raised in the Animal Experiment Center of Zhejiang Chinese Medical University, all animal experiments also took place at there, and the experimental animal license number was SYXK (Zhejiang) 2018–0012. The animal experiments were approved by the Experimental Animal Ethics Committee of Zhejiang Chinese Medical University (IACUC-20200506-04), and the experimental procedures complied with the relevant animal ethics guidelines.

### 2.2. Drugs and Reagents

Mesalazine was purchased from Shanghai Haoyuan Biomedical Technology Co., Ltd. (Shanghai, China); TNBS and formic acid were purchased from Sigma-Aldrich (St. Louis, MO, USA); methanol and acetonitrile were purchased from CNW Technologies (Shanghai, China); 2-chloro-L-phenylalanine was purchased from Shanghai Hengbai Biotech Co., Ltd. (Shanghai, China); and enzyme-linked immunosorbent assay kits were purchased from Nanjing Jiancheng Bioengineering Institute (Nanjing, Jiangsu, China).

### 2.3. Preparation of SLBZS

SLBZS is composed of 10 kinds of herbs (purchased from Hangzhou Binjiang Chinese Medicine Clinic of Zhejiang Chinese Medical University, Hangzhou, China), including Ren Shen, Fu Ling, Bai Zhu, Shan Yao, Bai Bian Dou, Lian Zi, Gao Cao, Yi Yi Ren, Jie Geng, and Sha Ren, and the composition details are shown in Table 1. The daily dose of SLBZS in adults is 111 g. According to the conversion ratio of surface area between rat and humans, the daily dose for rats was calculated to be 12 g/kg. This dose was used as the medium dose. We selected the high doses of SLBZS (24 g/kg) based on the previous studies of others and the experimental results of our previous pre-experiment [15–18]. Decoction preparation involved soaking the medicine in cold water for 30 min, boiling and simmering over a slow fire for 30 min, and decocting three times. Then, the medicines were concentrated to a final concentration of 1.0 g·mL^−1^. Part of the SLBZS liquid was stored at −80°C for ultra-performance liquid chromatography tandem mass spectrometry (UPLC-QTOF-MS) analysis. Each Chinese medicine was confirmed to be authentic.

### 2.4. UPLC-QTOF-MS Analysis

The samples of SLBZS were thawed in ice water, vortexed for 30 s, and centrifuged at 12,000 r at 4°C for 10 min. A 300 *μ*l aliquot of each individual sample was precisely transferred to an Eppendorf tube. After the addition of 1,000 *μ*l extraction solution (methanol: water = 4 : 1, v/v, including internal standard (IS) at a ratio of 1,000 : 10), all samples were vortexed for 30 s, sonicated for 10 min in an ice-water bath, incubated at −80°C for 1 h, and centrifuged at 12,000 r at 4°C for 10 min. A 400 *μ*l aliquot of the supernatant was passed through a 0.22 *μ*m filter membrane and then transferred to an autosampler vial for UPLC-MS/MS analysis, which was performed on an Agilent ultra-high performance liquid chromatography 1290 UPLC system with a Waters UPLC BEH C18 column (1.7 *μ*m, 2.1*∗*100 mm). The flow rate was set at 0.4 mL/min, and the sample injection volume was set at 5 *μ*L. The mobile phase consisted of 0.1% formic acid in water (A) and 0.1% formic acid in acetonitrile (B). The multistep linear elution gradient program was as follows: 0–3.5 min, 95–85% A; 3.5–6 min, 85–70% A; 6–6.5 min, 70–70% A; 6.5–12 min, 70–30% A; 12–12.5 min, 30–30% A; 12.5–18 min, 30–0% A; 18–25 min, 0–0% A; 25–26 min, 0–95% A; and 26–30 min, 95–95% A. A Q Exactive Focus mass spectrometer coupled with Xcalibur software was employed to obtain the MS and MS/MS data based on the IDA acquisition mode. During each acquisition cycle, the mass range was from 100 to 1500, the top three of every cycle were screened, and the corresponding MS/MS data were further acquired, such as sheath gas flow rate: 45 Arb, aux gas flow rate: 15 Arb, capillary temperature: 400°C, full ms resolution: 70000, MS/MS resolution: 17500, collision energy: 15/30/45 in NCE mode, and spray voltage: 4.0 kV (positive) or −3.6 kV (negative) [19].

### 2.5. Animal Model Establishment and Treatment Procedures

After the 7 days of adaptation period (rats were housed in a cage at 22 ± 2°C with a 12 h light/dark cycle and given sterile food and water), 45 rats were randomly divided into five groups: a normal control (NC) group, a model group, an SLBZS group, a mesalazine (Mes) group, and an SLBZS combined with mesalazine (SMes) group. Each group of nine rats was housed in one cage. Before modeling UC, we first constructed a rat model of spleen deficiency and dampness. The rats in the NC group were fed with normal-temperature saline 2 mL once a day; the rats in the other four groups were placed in a 2 cm pool every day from 8 : 00 to 16 : 00 and controlled sleep time for 8 h. In addition, on odd days, the rats were fed with 4°C saline 2 mL and fasted for 12 h, and on even days, the rats were fed with adequate feed and lard oil 4 mL [20]. A rat model of spleen deficiency and dampness was, thus, established by exposing to these conditions for 20 consecutive days.

On the 21st day, all rats were fasted for 24 h, with free access to water. UC was induced in rats in the model and medication treatment groups on the 22nd day. After intraperitoneal anesthesia with 3% pentobarbital sodium (0.15 mL/100 g), a urinary catheter was lubricated with paraffin oil and inserted 8 cm into the upper anus of the rat. Next, according to the dose of 100 mg/kg of rat, 5% TNBS (dissolved in 50% ethanol, 1 : 1 ratio) was injected into the urinary catheter, which was then placed flat for 2 min. The tail of the rat was then lifted and elevated for 1 min to ensure that the reagent fully penetrated into the intestinal cavity [21, 22]. The rat was placed in the cage and observed until it was fully awake and freely eating and drinking water. The day after the induction of UC, rats in the SLBZS group were given 24 g/kg SLBZS by intragastric gavage every day, rats in the Mes group received 0.3 g/kg mesalazine [23, 24], rats in the SMes group received 24 g/kg SLBZS and 0.3 g/kg mesalazine, and rats in the NC group and model group received an equal volume of normal saline. The above operations are repeated for all groups every day for a total of 14 days.

### 2.6. Animal Observations

General physical signs (coat color, mental state, mood, diet, sleep, and stools) were observed, and symptoms, such as diarrhea and prolapse, sticky stools, weight loss, lethargy, and decreased response, were recorded every day during the entire course of the experiment. Animals were weighed every day.

### 2.7. Evaluation Criteria of Spleen Deficiency and Dampness Syndrome

On the last day of modeling, the rats were scored for spleen deficiency and dampness syndromes. The total score was calculated as the sum of the mental state, activity state, coat condition, eye condition, stool color, stool consistency, and anal condition (Table 2) [25–27].

### 2.8. Disease Activity Index (DAI)

The severity of colitis was assessed daily by using a DAI scoring system, which scores for body weight loss, stool consistency, and rectal bleeding. The total score was calculated as (weight loss score + stool consistency score + rectal bleeding score)/3 (Table 3) [28].

### 2.9. Histological Analysis

After the experiment was completed, the rats were euthanized using CO_2_, and the colon was removed and measured. Then, the intestinal tissue was immediately fixed in 4% paraformaldehyde, embedded in paraffin, sectioned, subjected to hematoxylin and eosin (H&E) staining, and examined under light microscopy. The scoring criteria were used to determine the histopathological score, which was calculated as the sum of inflammation severity, crypt injury, and ulcer severity scores (Table 4) [29, 30].

### 2.10. Enzyme-Linked Immunosorbent Assay (ELISA)

After two weeks of drug treatment, the blood of the rats was collected and centrifuged at 4°C, 3,000 r/min for 10 min, and the supernatant was collected and stored in the refrigerator at −80°C for later use. This protocol was followed according to the ELISA kit instructions, in order to determine the levels of IL-1*β* and TNF-*α* in serum.

### 2.11. Bacterial DNA Extraction and 16S rDNA Gene Sequencing

After two weeks of drug treatment, rat feces were collected and placed in sterile centrifuge tubes, snap-frozen in liquid nitrogen, and stored at −80°C for later use. The E.Z.N.A.® Stool DNA Kit (Omega Bio-Tek, Norcross, GA, U.S.A) was used to extract microbial community genomic DNA. The DNA extract was checked on 1% agarose gel, and DNA concentration and purity were determined with NanoDrop 2000 UV-Vis spectrophotometer (Thermo Scientific, Wilmington, USA). The hypervariable region V3-V4 of the bacterial 16S rDNA gene was amplified with primer pairs 338F (5′-ACTCCTACGGGAGGCAGCAG -3′) and 806R (5′-GGACTACHVGGGTWTCTAAT -3′) by an ABI GeneAmp® 9700 PCR thermocycler (ABI, CA, USA). The PCR amplification of 16S rDNA gene was performed as follows: initial denaturation at 95°C for 3 min, followed by 27 cycles of denaturing at 95°C for 30 s, annealing at 55°C for 30 s and extension at 72°C for 45 s, single extension at 72°C for 10 min, and end at 4°C. The PCR mixtures contain 5 × TransStart FastPfu buffer 4 *μ*L, 2.5 mM dNTPs 2 *μ*L, forward primer (5 *μ*M) 0.8 *μ*L, reverse primer (5 *μ*M) 0.8 *μ*L, TransStart FastPfu DNA Polymerase 0.4 *μ*L, template DNA 10 ng, and finally ddH2O up to 20 *μ*L. PCR reactions were performed in triplicate. The PCR product was extracted from 2% agarose gel and purified using the AxyPrep DNA Gel Extraction Kit (Axygen Biosciences, Union City, CA, USA) according to manufacturer's instructions and quantified using Quantus™ Fluorometer (Promega, USA). Purified amplicons were pooled in equimolar and paired-end sequenced on an Illumina MiSeq PE300 platform/NovaSeq PE250 platform (Illumina, San Diego, USA) according to the standard protocols by Majorbio Bio-Pharm Technology Co., Ltd. (Shanghai, China).

### 2.12. Statistical Processing

SPSS software (version 25.0, SPSS Inc., Chicago, IL, USA) was used for data analysis. The data obtained were expressed as the average ± standard deviation (*X* ± SD). The independent sample *t*-test was used to compare data from the two groups, the ANOVA statistical test was performed to compare the data between multiple groups, when the data were normal distribution, and the nonparametric test was used when the data were non-normal distribution. *P* < 0.05 was considered statistically significant.

## 3. Results

### 3.1. Component Analysis of SLBZS

**Figure 1:**
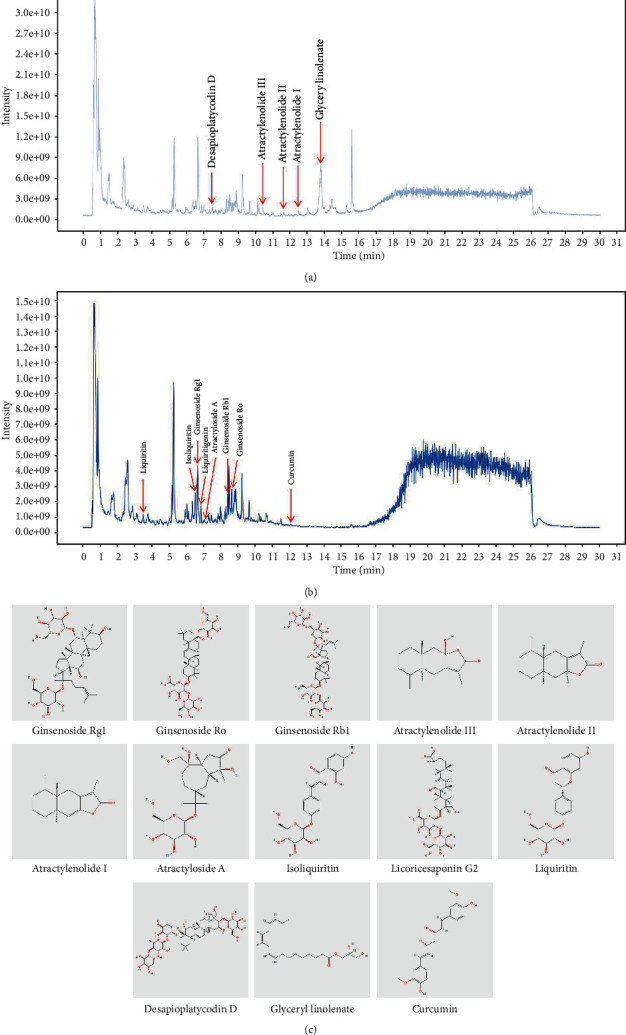
Identification of chemical components of SLBZS. (a) Total ion chromatography in positive ion modes for SLBZS samples; (b) total ion chromatography in negative ion modes for SLBZS samples; and (c) molecular structure of constituents.

To identify the major chemical components, SLBZS samples were analyzed by UHPLC-MS/MS. The total positive (Figure 1(a)) and negative (Figure 1(b)) ion chromatograms of SLBZS demonstrated the chemical composition of all compounds. Several components were found in SLBZS. As shown in Figure 1(c), thirteen compounds were distinguished: (1) ginsenoside Rg1, (2) ginsenoside Ro, (3) ginsenoside Rb1, (4) atractylenolide III, (5) atractylenolide II, (6) atractylenolide I, (7) atractyloside A, (8) isoliquiritin, (9) licoricesaponin G2, (10) liquiritin, (11) desapioplatycodin D, (12) glyceryl linolenate, and (13) curcumin.

### 3.2. General Behavior Indices

Following the establishment of a spleen deficiency and dampness model in rats, the activity of the rats decreased, aggressive and confrontational behaviors reduced, stools became soft or loose, hair withered and became dull, and the anus became red and swollen.

After the induction of UC, the rats appeared lethargic, hunched, frequently had closed eyes, severe bloody and loose stools, which were sticky and foul smelling, and a red and swollen anus. After treatment, the activity of the rats gradually increased, the bloody stool improved, and the redness and swelling of the anus decreased.

### 3.3. Physical Signs

**Figure 2:**
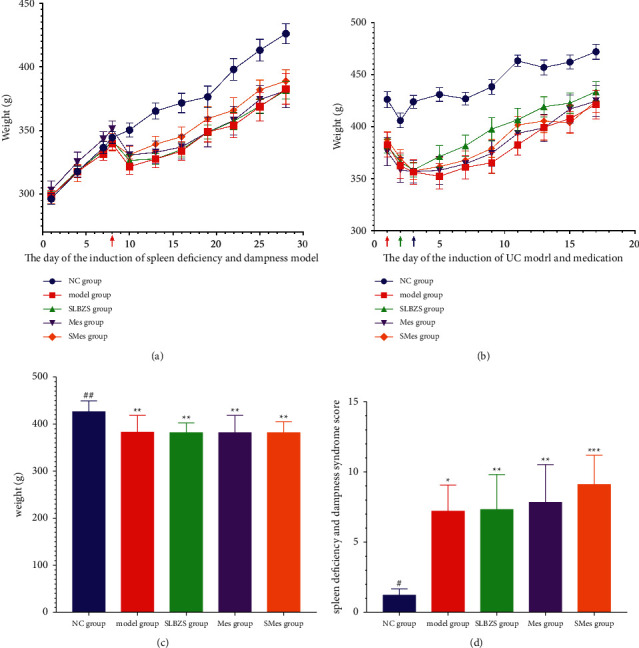
Physical observations and spleen deficiency and dampness syndrome scores. (a) Body weight changes in rats during establishing spleen deficiency and dampness model: the red arrow means the first day of spleen deficiency and dampness model creation; (b) body weight changes in rats during the medication period following ulcerative colitis (UC) induction: the red arrow indicates the beginning of fasting and fasted for 24 h, the green arrow means the day of UC model creation, and the blue arrow means the first day of medication; (c) body weight of rats after inducing spleen deficiency and dampness; and (d) spleen deficiency and dampness syndrome scores for rats in each group. ^*∗*^Compared with the NC group (*P* < 0.05); ^*∗∗*^compared with the NC group (*P* < 0.01); ^*∗∗∗*^compared with the NC group (*P* < 0.01); ^#^compared with the model group (*P* < 0.05); and ^##^compared with the model group (*P* < 0.05).

Before the induction of spleen deficiency and dampness, rats in the model and treatment groups did not exhibit a significant difference in body weight, compared to those in the NC group (*P* > 0.05). During the induction of a spleen deficiency and dampness model in rats, the body weights of rats in the model and treatment groups decreased in the early stage and gradually increased in the later stage (Figure 2(a)). On the last day of modeling, the weight of the rats in the model and treatment groups significantly differed from that of rats in the NC group (*P* < 0.01) (Figure 2(c)).

Since the rats were fasted for 24 hours on the 21st day, the weight of rats in each group decreased on the 22nd day. After intervention with TNBS, the weight of rats generally decreased at the beginning, and with the intervention of the drug, the weight of rats gradually increased (Figure 2(b)), but no significant difference was observed in the body weight of rats in each group after treatment (*P* > 0.05).

### 3.4. Evaluation and Score of Spleen Deficiency and Dampness Syndrome

On the 21st day, we found that the rats were lethargic and lazy, there was a reduction or disappearance of aggressive and confrontational behaviors of the rats, the stools of the rats were loose, the hair of the rats was dull, and the rats displayed a red and swollen anus. The animals in each group were scored for spleen deficiency and dampness syndromes. Compared with the NC group, the rats in the model, SLBZS, Mes, and SMes groups exhibited higher scores (*P* < 0.05; *P* < 0.01; *P* < 0.01; and *P* < 0.001) (Figure 2(d)).

### 3.5. DAI

**Figure 3:**
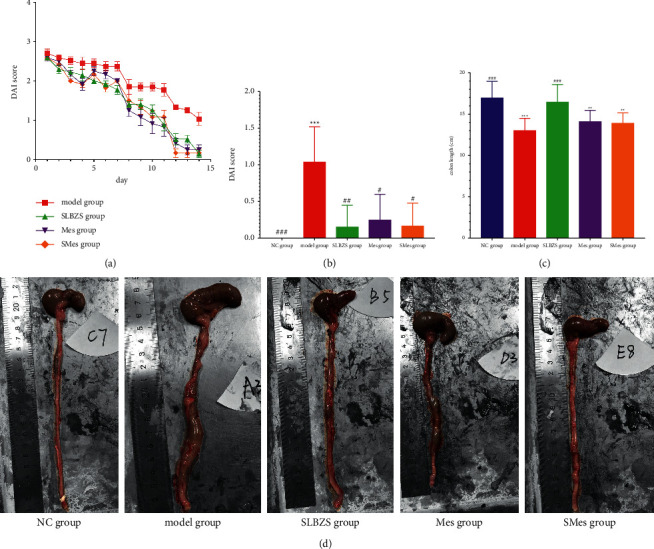
The DAI score, colon length, and morphology of rats in each group. (a) Change in DAI scores during the medication period following UC induction; (b) the DAI scores of rats in each group after receiving medication; (c) Comparison of the colon length in each group; and (d) typical colon anatomy of each group. ^*∗∗*^Compared with the NC group (*P* < 0.01); ^*∗∗∗*^compared with the NC group (*P* < 0.001); ^#^compared with the model group (*P* < 0.05); ^##^compared with the model group (*P* < 0.01); and ^###^compared with the model group (*P* < 0.001).

The DAI scores were recorded daily (Figure 3(a)). In the process of medication, the DAI score showed a gradually decreasing trend, and after 14 days of treatment, the DAI scores of rats in the NC, SLBZS, Mes, and SMes groups significantly differed from those of rats in the model group (*P* < 0.001; *P* < 0.01; *P* < 0.05; and *P* < 0.05) (Figure 3(b)).

### 3.6. Colon Length and Morphology

Whole colons from rats in the model group were significantly shorter than those from the NC group (*P* < 0.001). The colon length significantly increased following treatment with SLBZS, when compared to that observed in the model group (*P* < 0.001), and was not significantly different from that in the NC group (*P* > 0.05) (Figures 3(c) and 3(d)).

### 3.7. Histological Analysis

**Figure 4:**
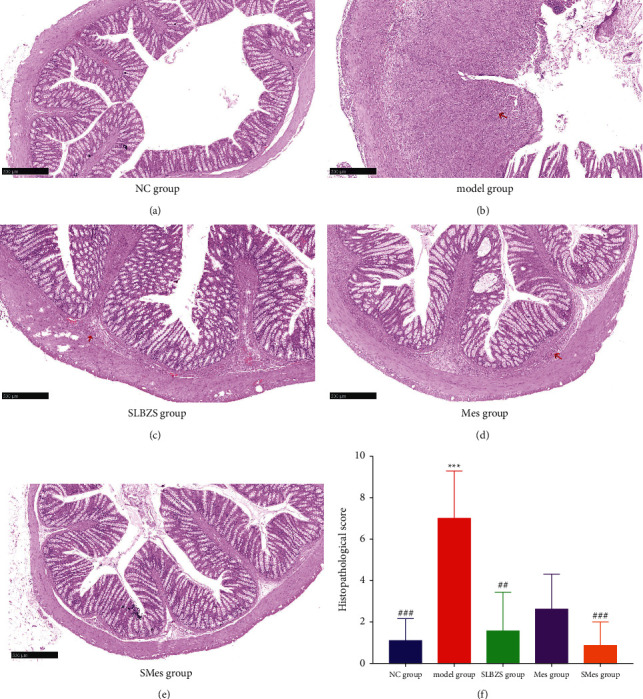
Histopathological sections from rat colons in each group and histopathological scores. (a) NC group; (b) model group; (c) SLBZS group; (d) Mes group; (e) SMes group; and (f) histopathological score of the colon in each group. The inflammatory cells were pointed out with red arrows. ^*∗∗∗*^Compared with the NC group (*P* < 0.001); ^###^compared with the model group (*P* < 0.001) ((a) to (e) all were magnified 5 times).

In the NC group, the structure of each layer of colon tissue was complete, the mucosal microvilli were neatly arranged, the goblet cells were abundant, crypt structure was normal, the colon glands were regular, and there were no inflammatory cell infiltrates. In the model group, there was evidence of mucosal epithelial cell necrosis, disordered cell arrangement, fewer goblet cells and crypts, and inflammatory cell infiltrates. In rats treated with SLBZS, mesalazine, or SLBZS combined with mesalazine, the structure of each cell layer was neat, the mucosal epithelial cell arrangement was slightly disordered but without shedding, few goblet cells and crypts were lost, and inflammatory cell infiltrates were reduced compared with those in the model group (Figures 4(a)–4(e)).

The histopathological scores of colons in the model group were significantly higher than those in the NC group (*P* < 0.001). The histopathological scores of the colon from rats in the SLBZS and SMes groups were significantly lower than those from rats in the model group (*P* < 0.01). There was no difference among the SLBZS, Mes, SMes, and the NC group (*P* > 0.05) (Figure 4(f)).

### 3.8. ELISA

**Figure 5:**
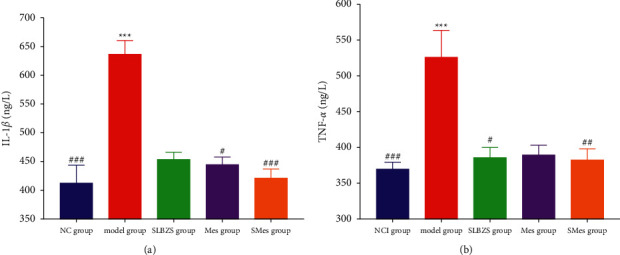
The level of inflammatory factor in serum of rats. (a) IL-1*β*; (b) TNF-*α*. ^*∗∗∗*^: compared with the NC group (*P* < 0.001); ^#^: compared with the model group (*P* < 0.05); ^##^: compared with the model group (*P* < 0.01); and ^###^: compared with the model group (*P* < 0.001).

Compared with the NC group, rats in the model group exhibited increased levels of IL-1*β* and TNF-*α* in serum (*P* < 0.001). Compared with that in the NC group, the levels of IL-1*β* and TNF-*α* did not significantly increase in the SLBZS group, Mes group, and SMes group (*P* > 0.05). Compared with the model group, rats in the SLBZS group exhibited lower levels of TNF-*α*(*P* < 0.05) in serum (Figure 5).

### 3.9. Intestinal Flora

#### 3.9.1. Sequencing Quality and Flora Diversity

**Figure 6:**
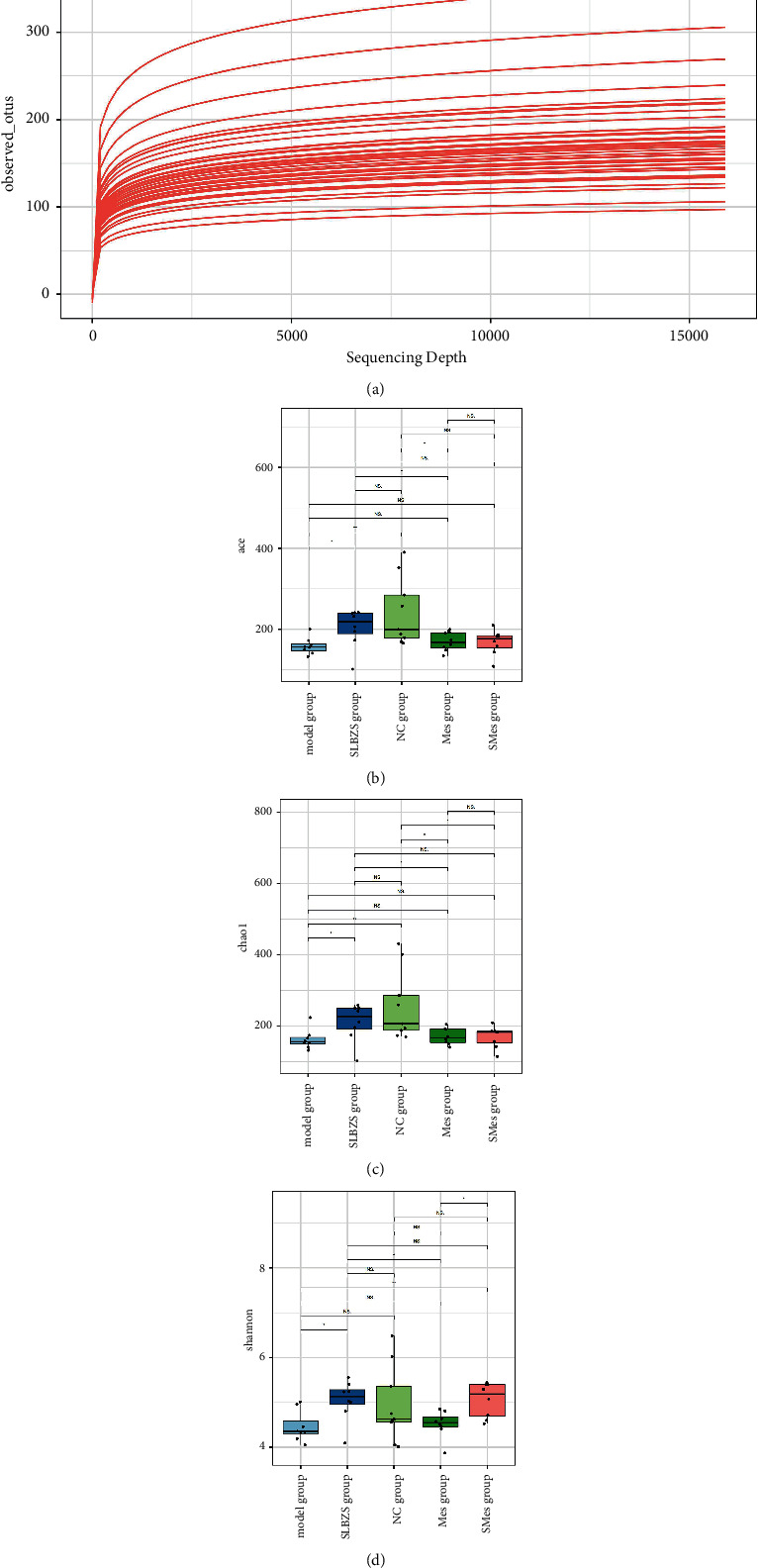
Sequencing quality and flora diversity analysis. (a) Dilution curve; (b) comparison of the ACE index for each group; (c) comparison of the Chao1 index for each group; and (d) comparison of the Shannon index for each group.

After clustering by dividing amplicon sequence variants (ASVs), a certain amount of sequencing data were randomly selected from the samples to generate a dilution curve and to evaluate the species abundance (Figure 6(a)). As the amount of sequencing increased, the curve obtained for each sample gradually flattened, indicating that the amount of sequencing data was reasonable and that increasing the amount of data would lead to only a small number of new species being identified. This suggests that the amount of sequencing used can reflect the total number of ASVs in the sample.

Alpha diversity analysis showed that the ACE and Chao1 indices of the model group were significantly lower than that of the NC group (*P* < 0.05) and were significantly higher in the SLBZS group than in the model group (*P* < 0.05). Compared with those in the model group, the ACE and Chao1 indices were higher in the Mes group and the SMes group, but the difference was not significant (*P* > 0.05) (Figures 6(b) and 6(c)). Compared with the NC group, the Shannon index in the model group was lower, but the difference was not significant (*P* > 0.05). The Shannon index in the SLBZS group (*P* < 0.05) and the SMes group (*P* < 0.01) was significantly higher than that of the model group, but there was no significant difference between the model group and the Mes group (*P* > 0.05) (Figure 6(d)).

#### 3.9.2. Comparative Analysis of Intestinal Flora Samples

**Figure 7:**
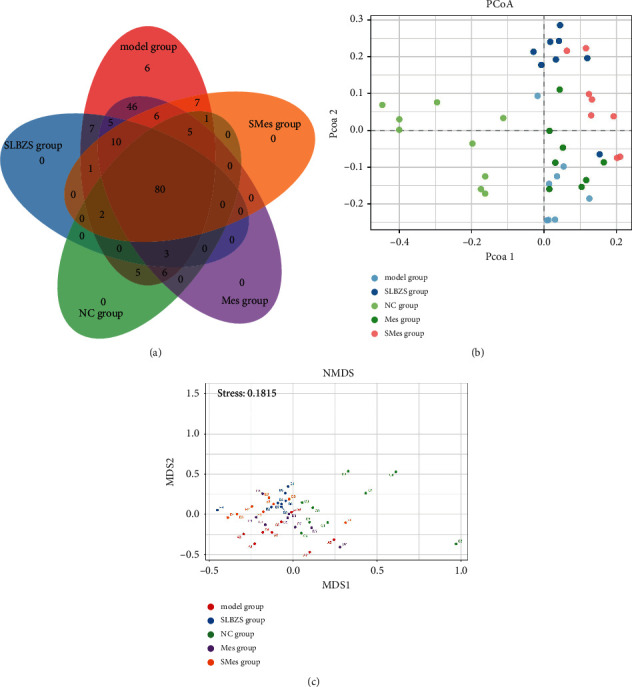
Comparative analysis of intestinal flora samples. (a) Venn diagram; (b) principal coordinate analysis (PCoA) chart; and (c) nonmetric multidimensional scaling analysis (NMDS) chart.

The Venn diagram in Figure 7(a) shows that the five groups share 80 ASVs; the NC and model groups share 96 ASVs; the NC group and the SLBZS group share 85 ASVs; and the SLBZS group and the model group share 108 ASVs. Beta diversity was then used to reflect the distribution of the different groups of intestinal flora. As shown in Figure 7(b), principal coordinate analysis (PCoA) did not reveal a significant overlap between the NC group and the other groups. The samples in the SLBZS group were relatively clustered, and the differences within the groups were small. The distance between the Mes group and the model group was relatively small, the overlap was large, and the difference between groups was small. Then, the nonmetric multidimensional scaling (NMDS) analysis was used to reflect the degree of differences and similarities between samples through the distance between points. As shown in Figure 7(c), the distance between samples in each group was small, and the difference in the flora was small. However, the distance between groups was large, indicating a noteworthy difference in the composition of the flora between groups.

**Figure 8:**
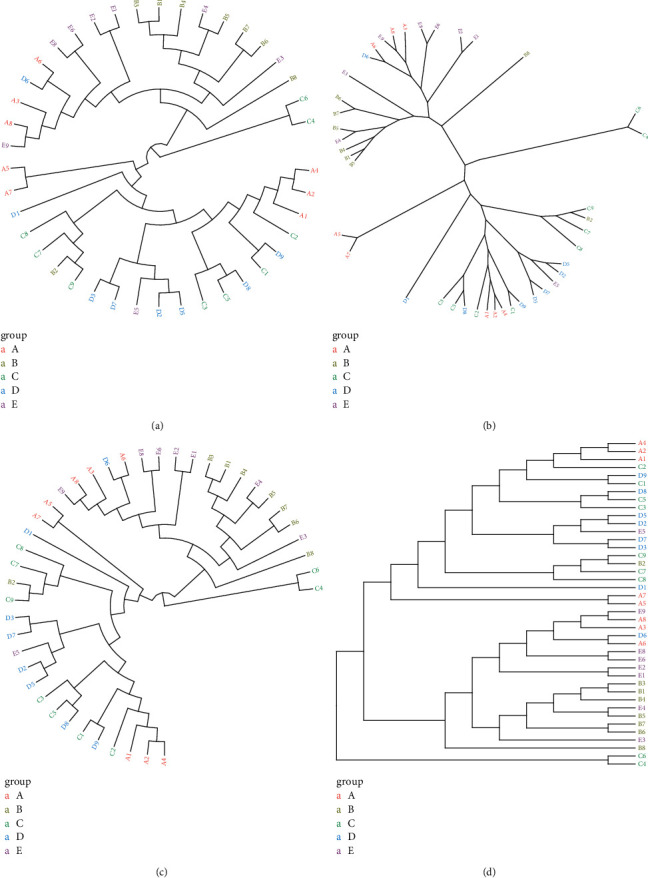
Comparative analysis of intestinal flora samples. Cluster tree analysis of the intestinal flora structure in each group. A: Model group; B: SLBZS group; C: NC group; D: Mes group; and E: SMes group.

The cluster tree analysis showed that there were a few differences in the intestinal flora between different groups. The intestinal floras of rats in the SLBZS group and the SMes group were similar. The intestinal floras of rats in the Mes and NC groups were relatively similar, as shown in Figure 8.

#### 3.9.3. Species Annotation and Differences in Intestinal Flora

Experimental results revealed that in the NC group, at the phylum level, the abundance of Firmicutes, Bacteroides, and Actinomycetes successively decreased, and the total amount accounted for 98.69% of all floras. At the genus level, *Blautia*, *Fusicatenibacter*, *Allobaculum*, *Ruminococcaceae* UCG-014 of Firmicutes, *Ruminococcaceae* UCG-008 of Firmicutes, *Subdoligranulum*, *Collinsella* of Actinomycetes, *Bifidobacterium* of Actinomycetes, *Lactobacillus* of Firmicutes, *Prevotella* 9, *Faecalibaculum* of Firmicutes, and CHKCI002 were the top 12 most-abundant bacterial genera, with abundances ranging from 0.07 to 36.98%.

In the model group, at the phylum level, the abundance of Firmicutes increased (*P* < 0.01), that of Bacteroides decreased (*P* < 0.05), and the abundance of Actinomycetes slightly decreased compared to those in the NC group, but the difference was not significant. At the genus level, the abundance of *Allobaculum*, *Bifidobacterium*, *Prevotella* 9, *Subdoligranulum, Ruminococcaceae* UCG-008, and *Ruminococcaceae* UCG-014 decreased compared with that of the NC group, although only the decrease in the *Subdoligranulum* was significant (*P* < 0.05). The abundance of *Blautia*, CHKCI002, Collinsella, Faecalibaculum, *Fusicatenibacter*, and *Lactobacillus* increased.

**Figure 9:**
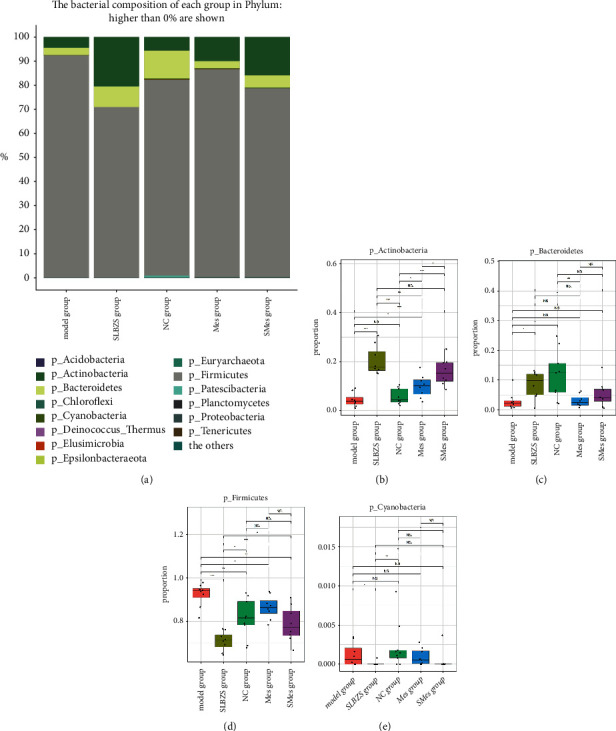
Species annotation and differences in intestinal flora at the phylum level in each group. (a) Histogram depicting the type and abundance of intestinal flora. (b) Abundance of Actinomycota, (c) Bacteroides, (d) Firmicutes, and (e) Cyanobacteria in each group.

**Figure 10:**
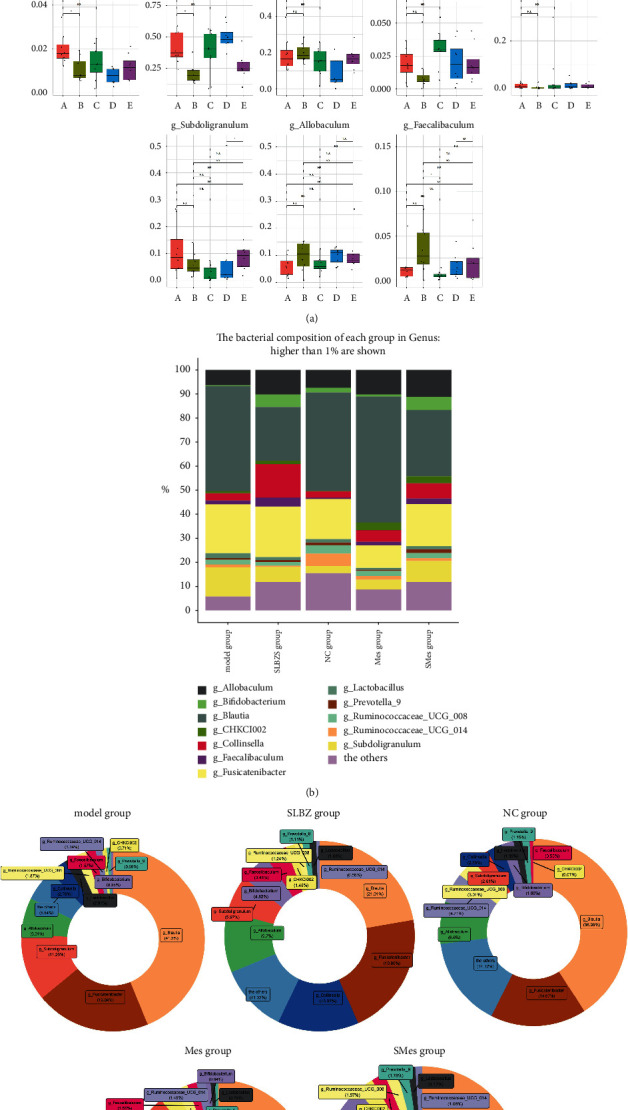
Species annotation and differences in intestinal flora at the genus level in each group. (a) Comparison of the main flora in the intestinal tract. (A) Model group; (B) SLBZS group; (C) NC group; (D) Mes group; and (E) SMes group. (b) Histogram and (c) pie chart depicting the types and abundance of intestinal flora.

**Figure 11:**
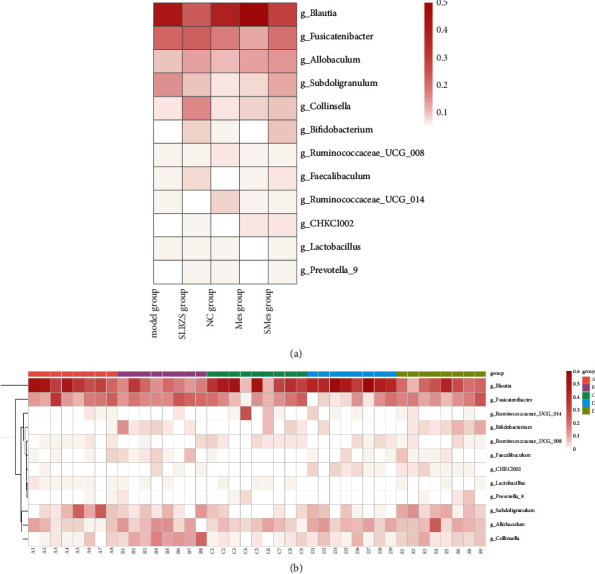
Species annotation and differences in intestinal flora at the genus level in each group. (a) Heat map comparing the main flora in the intestinal tract. (b) Comparison of the main flora in the intestinal tract. A: Model group; B: SLBZS group; C: NC group; D: Mes group; and E: SMes group.

In the SLBZS group, at the phylum level, the abundance of Actinobacteria and Bacteroides was higher (*P* < 0.01) while that of Firmicutes (*P* < 0.001) and Cyanobacteria (*P* < 0.05) was lower, compared with that in the model group. At the genus level, the abundance of *Allobaculum*, *Bifidobacterium*(*P* < 0.05), CHKCI002 (*P* < 0.05), *Collinsella*(*P* < 0.05), *Faecalibaculum*, *Prevotella* 9, *Fusicatenibacter*, and *Subdoligranulum* increased, while that of *Blautia*(*P* < 0.05), *Lactobacillus*(*P* < 0.05), *Ruminococcaceae* UCG-008, and *Ruminococcaceae* UCG-014 decreased (Figures 9–11).

**Figure 12:**
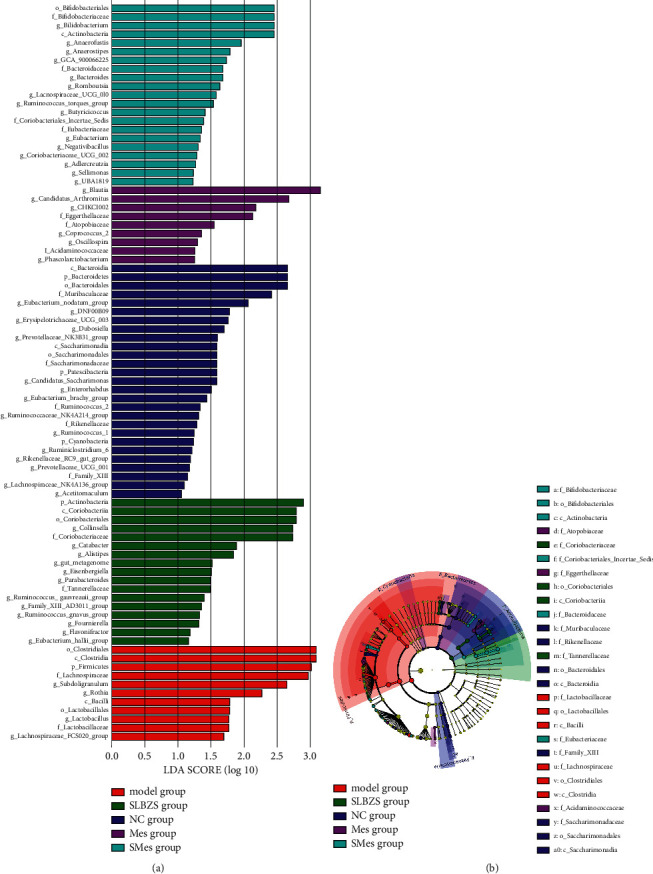
Annotation and difference analysis of intestinal flora structure. (a) Linear discriminant analysis (LDA) histogram; (b) evolutionary branch tree diagram.

The linear discriminant analysis effect size (LEfSe) results showed that Bacteroidetes, Bacteroidia, Bacteroidales, Muribaculaceae, and *Eubacterium nodatum group* were most abundant in the NC group; Firmicutes, Clostridia, Clostridiales, Lachnospiraceae, *Rothia*, and *Subdoligranulum* were most abundant in the model group. Actinobacteria, Coriobacteriia, Coriobacteriales, Coriobacteriaceae, *Collinsella*, and *Acetitomaculum* were most abundant in the SLBZS group (Figure 12).

#### 3.9.4. Prediction of Biological Function of Intestinal Flora

**Figure 13:**
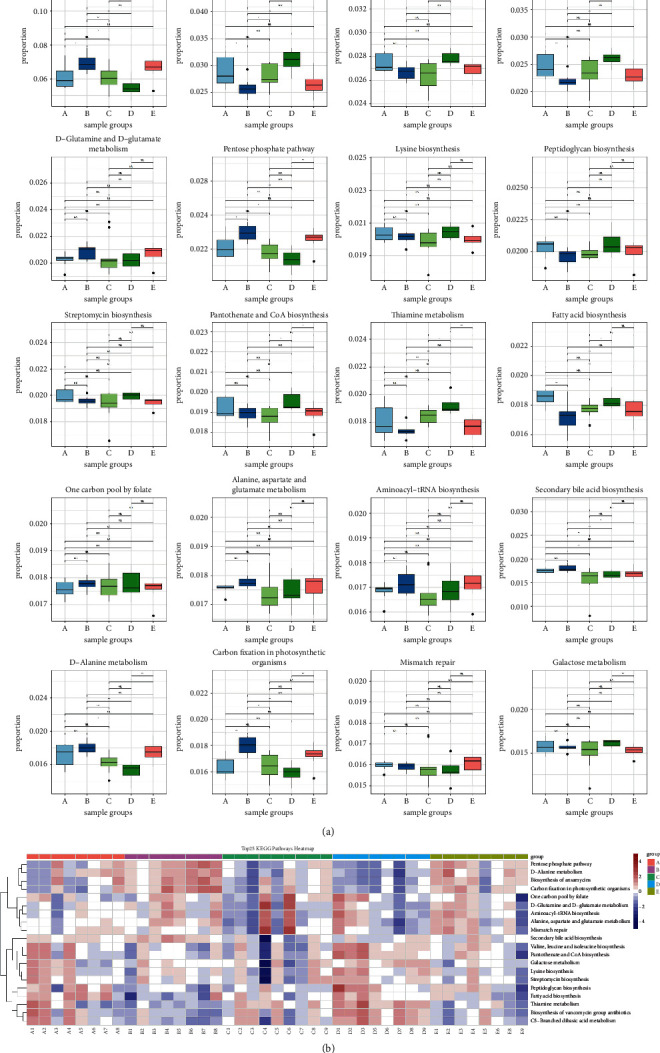
Prediction of biological function of intestinal flora. (a) Comparison of the top 20 KEGG pathways predicted between groups. A: Model group; B: SLBZS group; C: NC group; D: Mes group; and E: SMes group. (b) Heat map comparing the top 20 Kyoto Encyclopedia of Genes and Genomes (KEGG) pathways predicted.

To characterize the distinct functions of intestinal flora, we performed the PICRUSt analysis combined with the Kyoto Encyclopedia of Genes and Genomes (KEGG) analysis. As shown in Figure 13, fatty acid synthesis significantly increased after UC modeling (*P* < 0.05) and significantly decreased after SLBZS treatment (*P* < 0.001). There was no significant change in ansamycin synthesis after UC modeling; however, ansamycin synthesis increased after SLBZS treatment (*P* < 0.05). There was no significant change in D-glutamine and D-glutamate metabolism after UC modeling, and though this increased after SLBZS treatment, the difference was not significant. There was no significant change in the metabolism of pentose phosphate after UC modeling, and this significantly increased after SLBZS treatment (*P* < 0.05).

## 4. Discussion

TCM is characterized as a multicomponent and multitarget treatment. SLBZS is a commonly used prescription for spleen deficiency and dampness, many clinical and experimental studies have reported that SLBZS can inhibit inflammation, improve intestinal mucosal barrier function, improve intestinal immune regulation, and effectively treat UC [31–33]. In this study, we established a rat model of UC with spleen deficiency and dampness, and a rat model of spleen deficiency and dampness was established before UC induction. This resulted in rats that were suitable for treatment with SLBZS. Here, we determined that SLBZS was effective in UC rats with spleen deficiency and dampness. SLBZS was found to improve the symptoms and signs of UC rats with spleen deficiency and dampness, increase the length of the colon, and reduce pathological damage. These were consistent with previous research [15–18]. In addition, SLBZS was found to reduce the levels of inflammatory cytokines in serum, which suggests that SLBZS has an anti-inflammatory effect in the treatment of UC.

In this study, 16S rDNA high-throughput sequencing technology was used to study the effect of SLBZS on the intestinal flora of UC rats. The intestinal flora of the UC rats was disordered, which mainly manifested as a decrease in abundance and diversity, and with changes in flora structure and function. Further analysis revealed that, at the phylum level, the abundance of Actinobacteria and Bacteroides in the intestine of UC rats was lower than rats in the NC group, whereas the abundance of Firmicutes was higher than that in the intestine of rats in the NC group, which was not consistent with the results of previous studies [34, 35]. This difference may be attributed to the fact that in our study, the model group comprised rats with UC and a type of spleen deficiency and dampness, based on the theory of TCM. Following intervention with SLBZS, the abundance of Actinomycetes and Bacteroides in the rat intestine increased while that of Firmicutes was decreased to less than that in the NC group. SLBZS significantly increased the abundance of Actinomycetes in the intestine of UC rats, which is consistent with the results of previous studies [36]. Therefore, we speculate that SLBZS may improve the composition and function of the intestinal flora in UC rats by regulating the abundance of Actinomycota and its subordinate bacteria, thereby ameliorating mucosal damage and reducing inflammation.

At the genus level, the abundance of *Bifidobacterium* and *Allobaculum* in the intestine of UC rats was lower than that in the intestine of rats in the NC group. Following intervention with SLBZS, the abundance of *Bifidobacterium* and *Allobaculum* in the intestine of rats increased. *Bifidobacterium* is a probiotic that improves host health by stimulating the immune system and inhibiting pathogen growth [37]. In this study, the abundance of *Bifidobacteria* decreased after UC modeling and increased after intervention with SLBZS, which is consistent with the results of JH. Tao et al. [38] indicate that SLBZS treats UC by regulating the abundance of *Bifidobacteria*. Currently, the role of *Allobaculum* in the occurrence and development of UC remains controversial. Studies have shown that *Allobaculum* can produce short-chain fatty acids to protect the intestinal barrier, thereby acting as a beneficial bacterium to inhibit the occurrence and development of UC [35]. However, some studies have also reported an increase in *Allobaculum* abundance after UC modeling and a decrease after electroacupuncture and moxibustion treatment [39]. It is unclear whether *Allobaculum* is a beneficial bacterium. In this study, the abundance of *Allobaculum* decreased after UC modeling and increased following intervention with SLBZS. Thus, *Allobaculum* may function as a beneficial bacterium in the context of UC development. Further studies are needed to confirm the role of *Allobaculum* in UC.

Based on changes in the structure of intestinal flora in UC rats, we performed further experiments and predicted that fatty acid synthesis increased after the induction of UC, whereas it decreased following intervention with SLBZS. Short-chain fatty acids (SCFAs) include acetate, propionate, and butyrate. Butyrate is the main energy source for colon cells and maintains the stability of the intestinal environment through anti-inflammatory effects [40]. There were many studies that proved the importance of SCFAs in the occurrence and development of UC [40–42] and were consistent with our research results. However, as we did not utilize metabolomics in this study, it is not clear how fatty acid synthesis was increased. We estimated that the levels of harmful fatty acids increased after UC modeling. Fatty acid synthesis tended to be normal following intervention with SLBZS, indicating that SLBZS may aid in the regulation of fatty acid biosynthesis; however, the specific mechanism needs to be determined. Rifamycin is an ansamycin antibiotic. Rifaximin, a rifamycin derivative, is an intestine-specific antibiotic that can antagonize the effect of TNF-*α* by activating the pregnane X receptor and has also been shown to inhibit the transport of bacteria to the mesenteric lymph nodes [43, 44]. In this study, the biosynthesis of ansamycin increased following intervention with SLBZS. Studies have shown that oral glutamine is effective against DSS-induced colitis and that glutamine inactivates cytosolic phospholipase A2 by rapidly inducing MAPK phosphatase [45]. Therefore, SLBZS may promote the growth of beneficial bacteria and inhibit the growth of harmful bacteria by promoting glutamine synthesis and metabolism to improve UC symptoms. While there was no significant change in pentose phosphate pathway metabolism in UC rats, this pathway was upregulated following treatment with SLBZS (Figure 12). Studies have indicated that NADPH produced by pentose phosphate metabolism can be used as an antioxidant, and ribose-5-phosphate can be used for nucleotide synthesis, indicating that SLBZS may promote the production of NADPH to exert antioxidant effects [46].

In this study, we found that compared with mesalazine, SLBZS can also have an anti-inflammatory effect, useful in treating UC. In regulating the intestinal flora, SLBZS is more effective than mesalazine. Mesalazine alone in the treatment of UC cannot regulate the intestinal flora, while SLBZS alone can increase the abundance and diversity of intestinal flora, and SLBZS combined with mesalazine can increase the diversity of intestinal bacteria. This suggested that in clinical work, we can use SLBZS in combination with mesalazine to better regulate the intestinal flora and control the development of UC.

## 5. Conclusions

This study provides a more comprehensive explanation for the efficacy of SLBZS in the context of UC treatment based on the intestinal microbiota. In conclusion, SLBZS improves the structure and function of the intestinal flora by adjusting the abundance and diversity in UC rats. However, SLBZS is a compound TCM with diverse ingredients and a complex mechanism of action, and its exact anti-UC components and detailed mechanisms of action are yet to be elucidated. Further experiments are needed, which utilize the TCM network analysis method to explore SLBZS targets in the treatment of UC at the molecular level. We will further investigate the screened differential flora and important pathways involved in the occurrence and development of UC to provide more reliable experimental data to support the efficacy of SLBZS in UC treatment.

## Figures and Tables

**Table 1:** Composition information of each Chinese herbal medicine in SLBZS.

Latin name	Chinese name	Adult daily dose of herb (g)	Composition ratio (%)
Panax ginseng C. A. Mey.	Ren Shen	15	5
Poria cocos (Schw.) Wolf.	Fu Ling	15	5
Atractylodes macrocephala Koidz.	Bai Zhu	15	5
Dioscorea opposita Tunb.	Shan Yao	15	5
Dolichos lablab L.	Bai Bian Dou	12	4
Nelumbo nucifera Gaertn.	Lian Zi	9	3
Glycyrrhiza uralensis Fisch. Ex DC.	Gao Cao	9	3
Coix lacryma-jobi L.	Yi Yi Ren	9	3
Platycodon grandiflorus (Jacq.) A. DC.	Jie Geng	6	2
Amomum villosum Lour.	Sha Ren	6	2

**Table 2:** Scoring criteria of spleen deficiency and dampness syndrome.

Observation indexes	0	1	2	3
Mental state	Full of energy and quick response	Slightly tired limbs and less quick response	Fatigue, squint	Lethargy, get together, and closed eyes
Active status	Lively	Decreased voluntary activity	Tiredness, lazy movement, curled up limbs, and slow movement	Laziness, aggressive, and confrontational behavior disappear
Coat	Shiny color	Dim color	Rough, not lush, and shed	Withered, dull color, and severe hair loss
Eyes	Stare	Eye fissure narrowing	Squint	Eyes closed and have secretions
Stool color	Color black	Brown	Tan	Yellow
Stool consistency	Dry and shaped	Sticky and stylish	Not shaped, but not diarrhea	Diarrhea, sticky, and foul smelling
Anus	Whitish	Slightly red	Redness	Redness and congestion, and dilated anus

**Table 3:** Criteria for disease activity index.

Weight loss (%)	Stool consistency	Rectal bleeding	Score
None	Normal	Normal	0
1–5	Loose stools	Occult bleeding	1
6–10			2
11–15	Diarrhea	Gross bleeding	3
>15			4

**Table 4:** Evaluation of pathological score.

Inflammation severity	Crypt injury	Ulcer severity	Score
Few inflammatory cells in the lamina propria	Complete crypts	No ulcers	0
Increased numbers of granulocytes in the lamina propria	Loss of one third	1 or 2 ulcers	1
Confluent of inflammatory cells extend to the submucosa	Loss of two thirds	3 or 4 ulcers	2
Transmural extension of inflammatory infiltration	The entire crypt is lost	Fusion or extensive ulcers	3
	Erosion changes on the epithelial surface		4
	Fusion erosion		5

## Data Availability

The data used to support the findings of this study are available from the corresponding author upon request.

## Abbreviations

SLBZS: Shenling Baizhu San
UC: Ulcerative colitis
TNBS: 2,4,6-Trinitrobenzene sulfonic acid
DAI: Disease activity index
H&E: Hematoxylin and eosin
ELISA: Enzyme-linked immunosorbent assays
IL-1*β*: Interleukin-1*β*
TNF-*α*: Tumor necrosis factor-*α*
PICRUSt: Phylogenetic investigation of communities by reconstruction of unobserved states
TCM: Traditional Chinese medicine
ASVs: Amplicon sequence variants
PCoA: Principal coordinate analysis
NMDS: Nonmetric multidimensional scaling analysis
UPGMA: Unweighted pair group method with arithmetic mean
LEfSe: Linear discriminant analysis effect size
LDA: Linear discriminant analysis
KEGG: Kyoto Encyclopedia of Genes and Genomes.

## References

[1] van der Sloot K. W. J., Amini M., Peters V., Dijkstra G., Alizadeh B. Z. (2017). Inflammatory bowel diseases: review of known environmental protective and risk factors involved. *Inflammatory Bowel Diseases*.

[2] Song Z.-M., Liu F., Chen Y.-M., Liu Y.-J., Wang X.-D., Du S.-Y. (2019). CTGF-mediated ERK signaling pathway influences the inflammatory factors and intestinal flora in ulcerative colitis. *Biomedicine & Pharmacotherapy*.

[3] Yuan Z., Yang L., Zhang X., Ji P., Wei Y. (2020). Therapeutic effect of n-butanol fraction of Huang-lian-Jie-du decoction on ulcerative colitis and its regulation on intestinal flora in colitis mice. *Biomedicine & Pharmacotherapy*.

[4] Rapport F., Clement C., Seagrove A. C., Alrubaiy L., Hutchings H. A., Williams J. G. (2019). Patient views about the impact of ulcerative colitis and its management with drug treatment and surgery: a nested qualitative study within the CONSTRUCT trial. *BMC Gastroenterology*.

[5] Xie J. R., Sun T., Zhang B. (2016). Treatment progress of UC with TCM and its relative advantages. *Liao Ning Journal of Traditional Chinese Medicine*.

[6] Gu S. Z., Xue Y., Zhang Y. L., Pan J., Tang Y. N., Dou D. B. (2018). Meta-analysis of the efficacy of oral traditional Chinese medicine in the treatment of ulcerative colitis. *Chinese Journal of Integrated Traditional and Western Medicine on Digestion*.

[7] Yue H., Wang T. F., Chen J. M. (2010). Modern literature of common syndromes and syndrome factors of ulcerative colitis. *Journal of Beijing University of Traditional Chinese Medicine*.

[8] Ding L. H., Jia Y. X., Cheng Y. X. (2018). Establishment of rat model of ulcerative colitis with spleen deficiency and dampness. *Journal of Chinese Clinical Medicine*.

[9] Li Z., Wang J., Cai R. L., Wang Y. W., Hu J. P. (2012). Establishment and evaluation of a rat model of ulcerative colitis with syndrome of dampness stagnancy due to spleen deficiency. *Journal of Chinese Integrative Medicine*.

[10] Zhang L. P., Xiao M. (2016). Long-term efficacy and safety of shenling Baizhu powder treating ulcerative colitis. *Liaoning Journal of Traditional Chinese Medicine*.

[11] Zhao W. G., Hou X. (2020). Clinical observation on treating ulcerative colitis of the Pixu Shizu type with Shenling Baizhu San plus mesalazine tablets. *Clinical Journal of Chinese Medicine*.

[12] Yang Y. (2018). Clinical observation on treatment of ulcerative colitis with shenlingbaizhu powder and mesalazine. *Chinese Journal of Ethnomedicine and Ethnopharmacy*.

[13] Lepage P., Häsler R., Spehlmann M. E. (2011). Twin study indicates loss of interaction between microbiota and mucosa of patients with ulcerative colitis. *Gastroenterology*.

[14] Ohkusa T., Yoshida T., Sato N., Watanabe S., Tajiri H., Okayasu I. (2009). Commensal bacteria can enter colonic epithelial cells and induce proinflammatory cytokine secretion: a possible pathogenic mechanism of ulcerative colitis. *Journal of Medical Microbiology*.

[15] Yu H. Q., Jia Y. X., Cheng Y. X. (2018). Effect of Shenlingbaizhu powder on the expression of IL-6, IL-10 and c-fos gene protein in colon tissue of UC rats with spleen deficiency and dampness. *Lishizhen Medicine and Materia Medica Research*.

[16] Wang J. J., Chi L., Wang W. J. (2019). Influence of Shenling Baizhu Powder on mRNA expression of NLRP3,NF-*κ*B,MUC2,TFF3 in rats of ulcerative colitis. *World Journal of Integrated Traditional and Western Medicine*.

[17] Ding L. H., Jia Y. X., Chen Y. X. (2018). Effect of shenling Baizhu san on expressions of IL-13, IL-23 and COX-2, CREB in ulcerative colitis rats with spleen deficiency and dampness. *Chinese Journal of Experimental Traditional Medical Formulae*.

[18] Zhou H., Zhang M., Wu F., Li H., Zhang S. (2019). Mechanism of Shenling Baizhu San in treating dextran sulfate sodium-induced colitis in rats. *Journal of Hainan Medical University*.

[19] Xuan C., Xi Y.-M., Zhang Y.-D., Tao C.-H., Zhang L.-Y., Cao W.-F. (2021). Yiqi jiedu huayu decoction alleviates renal injury in rats with diabetic nephropathy by promoting autophagy. *Frontiers in Pharmacology*.

[20] Li Z. H., Cai R. L., Luo M. X., Wang R. (2020). Effect of shenling baizhusan on protein and mRNA expression of NF-*κ*B p65, I*κ*B*α*, I*κ*K*β* in ulcerative colitis rats with syndrome of dampness stagnancy due to spleen deficiency. *Chinese Journal of Experimental Traditional Medical Formulae*.

[21] Eeckhaut V., Machiels K., Perrier C. (2013). Butyricicoccus pullicaecorumin inflammatory bowel disease. *Gut*.

[22] Xu B., Li Y.-l., Xu M. (2017). Geniposide ameliorates TNBS-induced experimental colitis in rats via reducing inflammatory cytokine release and restoring impaired intestinal barrier function. *Acta Pharmacologica Sinica*.

[23] Ahmad H., Verma S., Kumar V. L. (2018). Effect of roxithromycin on mucosal damage, oxidative stress and pro-inflammatory markers in experimental model of colitis. *Inflammation Research*.

[24] Pandey A., Verma S., Kumar V. L. (2017). Metformin maintains mucosal integrity in experimental model of colitis by inhibiting oxidative stress and pro-inflammatory signaling. *Biomedicine & Pharmacotherapy*.

[25] Zhang S. S., Hu L., Li R. L. (2017). Expert consensus on diagnosis and treatment of spleen deficiency syndrome in traditional Chinese medicine (2017). *Journal of Traditional Chinese Medicine*.

[26] Chen H., Liu Z. Y., Hou J. H. (2019). Establishment and objective evaluation of simple obesity animal model with spleen deficiency and dampness resistance. *Journal of Jiangxi College of Traditional Chinese Medicine*.

[27] Dong Y. X., Li T. H., Liu Y. (2020). Comparison of clinical syndromes of two spleen-qi deficiency syndrome models in rats. *Journal of Traditional Chinese Medicine University of Hunan*.

[28] Zong S.-y., Pu Y.-q., Xu B.-l., Zhang T., Wang B. (2017). Study on the physicochemical properties and anti-inflammatory effects of paeonol in rats with TNBS-induced ulcerative colitis. *International Immunopharmacology*.

[29] Yassin M., Kissow H., Vainer B. (2018). Cytoglobin affects tumorigenesis and the expression of ulcerative colitis-associated genes under chemically induced colitis in mice. *Scientific Reports*.

[30] Shin E. J., Sung M. J., Yang H. J., Kim M. S., Hwang J. T. (2014). Boehmeria nivea attenuates the development of dextran sulfate sodium-induced experimental colitis. *Mediators of Inflammation*.

[31] Quan L. Z., Tan J. H. (2017). Clinical study of shenling Baizhu san for ulcerative colitis. *Journal of New Chinese Medicine*.

[32] Jia Y. X., Bi D. Y., Duan Y. Q. (2018). Effect of shenling Baizhu powder on the colonic expression of p38MAPK, TNF-*α* and IL-4 in UC rats with spleen deficiency and dampness retention. *Acta Chinese Medicine and Pharmacology*.

[33] Xu Y. B., Chen X., Huang Y. Q. (2018). Clinical effects of the Shenling Baizhu Jianpi granule on chronic colitis and immune function. *Clinical Journal of Chinese Medicine*.

[34] Lee W.-T., Tung Y.-T., Wu C.-C., Tu P.-S., Yen G.-C. (2018). Camellia oil (camellia oleifera abel.) modifies the composition of gut microbiota and alleviates acetic acid-induced colitis in rats. *Journal of Agricultural and Food Chemistry*.

[35] Yu W., Su X., Chen W. (2019). Three types of gut bacteria collaborating to improve Kui Jie’an enema treat DSS-induced colitis in mice. *Biomedicine & Pharmacotherapy*.

[36] Sun Z., Pei W., Guo Y. (2019). Gut microbiota-mediated NLRP12 expression drives the attenuation of dextran sulphate sodium-induced ulcerative colitis by qingchang wenzhong decoction. *Evidence-Based Complementary and Alternative Medicine*.

[37] Liu Y., Wang X., Chen Q. (2020). Camellia sinensis and litsea coreana ameliorate intestinal inflammation and modulate gut microbiota in dextran sulfate sodium-induced colitis mice. *Molecular Nutrition & Food Research*.

[38] Tao J.-H., Duan J.-A., Jiang S., Feng N.-N., Qiu W.-Q., Ling Y. (2017). Polysaccharides from Chrysanthemum morifolium Ramat ameliorate colitis rats by modulating the intestinal microbiota community. *Oncotarget*.

[39] Wei D., Xie L., Zhuang Z. (2019). Gut microbiota: a new strategy to study the mechanism of electroacupuncture and moxibustion in treating ulcerative colitis. *Evidence-Based Complementary and Alternative Medicine*.

[40] Parada Venegas D., De la Fuente M. K., Landskron G. (2019). Short chain fatty acids (SCFAs)-Mediated gut epithelial and immune regulation and its relevance for inflammatory bowel diseases. *Frontiers in Immunology*.

[41] Zhuang X., Li T., Li M. (2019). Systematic review and meta-analysis: short-chain fatty acid characterization in patients with inflammatory bowel disease. *Inflammatory Bowel Diseases*.

[42] Lloyd-Price J., Arze C., Arze C. (2019). Multi-omics of the gut microbial ecosystem in inflammatory bowel diseases. *Nature*.

[43] Sartor R. B. (2016). Review article: the potential mechanisms of action of rifaximin in the management of inflammatory bowel diseases. *Alimentary Pharmacology & Therapeutics*.

[44] Rosette C., Agan F. J., Rosette N. (2019). Rifamycin SV exhibits strong anti-inflammatory in vitro activity through pregnane X receptor stimulation and NF*κ*B inhibition. *Drug Metabolism and Pharmacokinetics*.

[45] Jeong S.-Y., Im Y., Youm J., Lee H.-K., Im S.-Y. (2018). l-glutamine attenuates DSS-induced colitis via induction of MAPK phosphatase-1. *Nutrients*.

[46] Cheung E. C., Athineos D., Lee P. (2013). TIGAR is required for efficient intestinal regeneration and tumorigenesis. *Developmental Cell*.

